# Risk score modeling of multiple gene to gene interactions using aggregated-multifactor dimensionality reduction

**DOI:** 10.1186/1756-0381-6-1

**Published:** 2013-01-08

**Authors:** Hongying Dai, Richard J Charnigo, Mara L Becker, J Steven Leeder, Alison A Motsinger-Reif

**Affiliations:** 1 Research Development and Clinical Investigation, Children’s Mercy Hospital, Kansas City, MO, 64108, USA; 2 Division of Clinical Pharmacology and Medical Toxicology, Department of Pediatrics, Children’s Mercy Hospital, Kansas City, MO, 64108, USA; 3 Department of Statistics, University of Kentucky, Lexington, KY, 40506, USA; 4 Bioinformatics Research Center, Department of Statistics, North Carolina State University, Raleigh, NC, 27695, USA

**Keywords:** A-MDR, Epistasis enriched risk score, Epistasis enriched gene network, pRR, pOR, pChi

## Abstract

**Background:**

Multifactor Dimensionality Reduction (MDR) has been widely applied to detect gene-gene (GxG) interactions associated with complex diseases. Existing MDR methods summarize disease risk by a dichotomous predisposing model (high-risk/low-risk) from one optimal GxG interaction, which does not take the accumulated effects from multiple GxG interactions into account.

**Results:**

We propose an Aggregated-Multifactor Dimensionality Reduction (A-MDR) method that exhaustively searches for and detects significant GxG interactions to generate an epistasis enriched gene network. An aggregated epistasis enriched risk score, which takes into account multiple GxG interactions simultaneously, replaces the *dichotomous* predisposing risk variable and provides higher resolution in the quantification of disease susceptibility. We evaluate this new A-MDR approach in a broad range of simulations. Also, we present the results of an application of the A-MDR method to a data set derived from Juvenile Idiopathic Arthritis patients treated with methotrexate (MTX) that revealed several GxG interactions in the folate pathway that were associated with treatment response. The epistasis enriched risk score that pooled information from 82 significant GxG interactions distinguished MTX responders from non-responders with 82% accuracy.

**Conclusions:**

The proposed A-MDR is innovative in the MDR framework to investigate aggregated effects among GxG interactions. New measures (pOR, pRR and pChi) are proposed to detect multiple GxG interactions.

## Background

Human diseases usually have complex inheritance patterns, and single nucleotide polymorphisms (SNPs) has been utilized to explain the variation in susceptibility to many common complex diseases as well as the response to drug therapy. The advancement of genotyping technology has made genotypic data readily accessible to investigators at low cost. However, many challenges remain with regard to identifying genes that render people susceptible to non-Mendelian disorders and in understanding the associations and functional relationships among genes. More and more, researchers have been advocating for advanced statistical analysis to quantify complex and interactive biological and genetic relationships [1,2].

Multifactor Dimensionality Reduction (MDR) is a statistical paradigm for characterizing and detecting nonlinear complex gene-to-gene interactions (epistasis) possibly associated with susceptibility to disease [3]. When numerous genes are involved in a complex canonical pathway or network, traditional approaches for data analysis, such as a Chi-square test or Fisher’s exact test, might not detect the associations between risk factors and outcomes since these approaches assess only marginal main effects of the identified risk factors. Although one can employ logistic regression or other standard multivariate categorical data analysis approaches to explore interactions among SNPs, there are an enormous number of possible interactions in a model with both linear and nonlinear effects. Consequently, standard multivariate categorical data analysis approaches might detect very few interactions, and even then the cost in terms of sample size might be immense. MDR addresses these difficulties by converting high-dimensional genotypic data into a single predictive variable. Genotypic combinations are used to define high risk and low risk strata for the one-dimensional predisposing risk factor. MDR can reveal non-linear epistasis at a moderate sample size with no requirements on the underlying distributions of genotypes or outcomes [4].

The most commonly used MDR approach is described in detail by Ritchie et al. [3]. To distinguish this method from its various extensions, we will refer to it as the original MDR method. Related statistical software has been developed by Hahn et al. [4], Bush et al. [5], Winham and Motsinger-Reif [6], and Moore and colleagues (http://www.epistasis.org). In general, the MDR process can also be combined with a filter preprocess step by first applying global testing and filtration techniques to select the optimal number of SNPs for MDR analysis by searching for a subset of genes likely to interact with other genes using the ReliefF filtering process [7,8].

Details of the MDR [3] are briefly described here. MDR performs an exhaustive search of all variables and variable combinations to identify univariate or multivariate disease risk models. For each locus or multi-locus combination, attribute construction is performed to make a single variable with two categories: high risk and low-risk. A genotype or combination of genotypes is assigned high risk status if the ratio of affected subjects to unaffected subjects exceeds a pre-determined threshold, and low-risk otherwise. This step consolidates the high-dimensional risk space into a one-dimensional predictive variable. Typically, a 5-fold or 10- fold cross-validation procedure is employed, beginning with the random division of the original data set into five or ten subsets of approximately equal sizes [9]. For 10-fold cross-validation, a model is fit using nine of the ten subsets (collectively referred to as training data), and then the model is applied to classify observations in both the training data and the tenth subset not used to fit the model (referred to as validation data). This entire process is repeated ten times, with one of the ten subsets acting as the validation data [10]. The model’s training accuracy and testing accuracy are defined as the percentage of correct classifications in the corresponding data sets. The optimal one-locus, two-locus, and three-locus MDR models with the highest testing accuracy are identified. A one-locus model estimates the main effect of each SNP, while multi-locus models investigate the interactions among relevant SNPs. The cross validation consistency (CVC) is the number of times in a 10-fold cross validation that a particular multifactorial combination is identified as an optimal model for the training data. Finally, statistical significance of the optimal models is assessed by 1000- or 10000-count permutation testing [11].

MDR has been applied to identify gene-gene interactions conferring susceptibility to common diseases, including hypertension [12], bladder cancer [13], Type 2 diabetes [14], and rheumatoid arthritis [15,16]. Several extensions of the MDR method have been proposed. These methods entail the use of odds ratios [17], log-linear methods [18], generalized linear models [19], methods for data highly imbalanced with the disease outcome [20], model-based methods [21], contingency table measures of classification accuracy [22] and familial data [23,24].

In the present work, we propose an Aggregated-Multifactor Dimensionality Reduction (A-MDR) method that exhaustively searches for statistically significant gene-gene (GxG) interactions to generate a gene interaction network. In particular, an epistasis enriched risk score replaces the traditional *dichotomous* predisposing risk factor in quantifying the degree of susceptibility to a disease. We also introduce and compare new GxG interaction measures (pOR, pRR and pChi). An adjustment for multiple comparisons is implemented to limit false positive discoveries. In the current study we introduce the new approach, evaluate its performance in a range of simulations, and apply it to a real dataset from Juvenile Idiopathic Arthritis patients treated with methotrexate (MTX).

## Method

**Figure 1:**
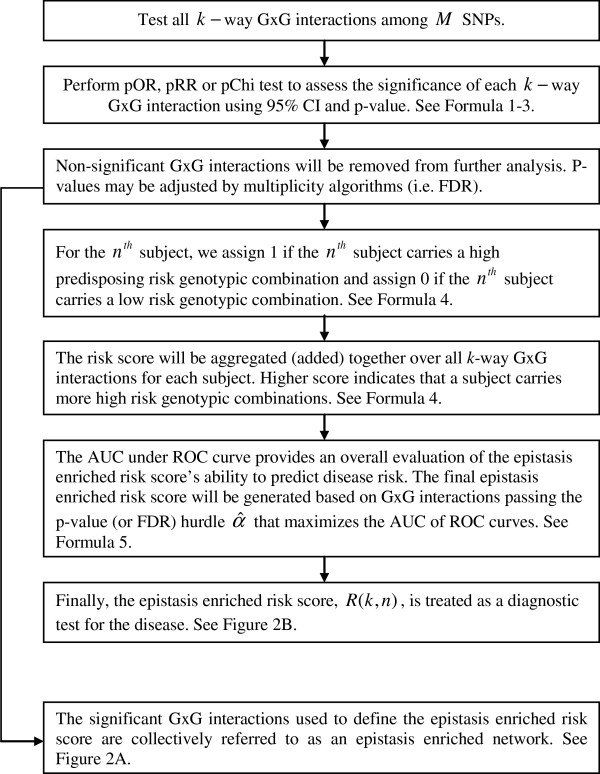
Flow chart of Aggregated-Multifactor Dimensionality Reduction (A-MDR).

The A-MDR method proposed herein can be applied to detect interactions among alleles, genotypes, and other categorical explanatory variables. Without loss of generality, we present the A-MDR method for SNPs with three common states (0 - homozygous reference, 1 - heterozygous, 2 - homozygous variant). The steps of the A-MDR are outlined in Figure 1.

### Detect multiple GxG interactions using the pOR, pRR or pChi test

The starting point for the A-MDR method is the construction of a predisposing risk factor. Suppose we want to investigate *k*–way GxG interactions among *M* SNPs. For one particular *k*–way GxG interaction, there are 3^*k*^ (*SNP*_(1)_ = 0, 1, 2 × *SNP*_(2)_ = 0, 1, 2 × ⋯ × *SNP*_(*k*)_ = 0, 1, 2) different genotypic combinations. Denote these 3^*k*^ genotypic combinations as *C*_*ij*_ where *j* = 1, 2, ⋯, 3^*k*^ stands for different genotypic combinations within one *k*–way GxG interaction. We need another subscript $i=1,2,\cdots ,\left(\begin{array}{l} M\\ k \end{array}\right)$ to cover all *k*–way GxG interactions among _*M*_ SNPs. Let *X*_*ij*_ and *Y*_*ij*_ be the numbers of affected (Test) and unaffected (Control) subjects in the *j*^*th*^ genotypic combination of the *i*^*th*^*k*–way GxG interaction. Let $p_{i0}=\left(\sum_{j=1}^{3^{k}}X_{\text{ij}}\right)/\left(\sum_{j=1}^{3^{k}}X_{\text{ij}}+\sum_{j=1}^{3^{k}}Y_{\text{ij}}\right)\in \left(0,1\right)$ be the threshold for disease risk above which a person is deemed highly susceptible using a Naïve Bayes classifier [25]. Genotypic combinations are then classified into high-risk predisposing risk groups and low-risk predisposing risk groups, *n*_11_*n*_12_*n*_21_*n*_22_, in Table 1 where *I*{_·_} denotes an indicator function.

**Table 1:** **2x2 Predisposing risk table (Subscript *i *is omitted for *n*’s.)**

	**Case**	**Control**	**Total**
High Predisposing Risk	$n_{11}=\sum_{j=1}^{3^{k}}X_{\text{ij}}I\left\{\frac{X_{\text{ij}}}{X_{\text{ij}}+Y_{\text{ij}}}>p_{i0}\right\}$	$n_{12}=\sum_{j=1}^{3^{k}}Y_{\text{ij}}I\left\{\frac{X_{\text{ij}}}{X_{\text{ij}}+Y_{\text{ij}}}>p_{i0}\right\}$	*n*_1+_
Low Predisposing Risk	$n_{21}=\sum_{j=1}^{3^{k}}X_{\text{ij}}I\left\{\frac{X_{\text{ij}}}{X_{\text{ij}}+Y_{\text{ij}}}\leq p_{i0}\right\}$	$n_{22}=\sum_{j=1}^{3^{k}}Y_{\text{ij}}I\left\{\frac{X_{\text{ij}}}{X_{\text{ij}}+Y_{\text{ij}}}\leq p_{i0}\right\}$	*n*_2+_
Total	*n*_+1_	*n*_+2_	*N*

We propose to perform one of the following measures to assess the *i*^*th*^*k*–way GxG interaction (the subscript *i* is omitted for *n*’s and *e*’s):

(a) the predisposing odds ratio (*pOR*),

(1) $$\text{pO}R_{i}=\frac{n_{11}n_{22}/\left(n_{12}n_{21}\right)}{F_{0}^{-1}\left(F\left(n_{11}n_{22}/\left(n_{12}n_{21}\right)\right)\right)}\text{;}$$

(b) the predisposing relative risk (pRR),

(2) $$\text{pR}R_{i}=\frac{\frac{n_{11}/\left(n_{11}+n_{12}\right)}{n_{21}/\left(n_{21}+n_{22}\right)}}{F_{0}^{-1}F\left(\frac{n_{11}/\left(n_{11}+n_{12}\right)}{n_{21}/\left(n_{21}+n_{22}\right)}\right)}\text{,}$$

and

(c) the predisposing chi-square (pChi) test statistic,

(3) $$\text{pCh}i_{i}=\frac{\sum_{s=1}^{2}\sum_{t=1}^{2}\frac{{\left(n_{\text{st}}-e_{\text{st}}\right)}^{2}}{e_{\text{st}}}}{F_{0}^{-1}F\left(\sum_{s=1}^{2}\sum_{t=1}^{2}\frac{{\left(n_{\text{st}}-e_{\text{st}}\right)}^{2}}{e_{\text{st}}}\right)}\text{,}$$

where $e_{\text{st}}=\frac{n_{s+}n_{+t}}{N}$ is the expected number of subjects in predisposing risk stratum *s* (1 = high predisposing risk, 2 = low predisposing risk) and disease stratum *t* (1 = Case, 2 = Control) under a null hypothesis of no association between the predisposing risk factor and the disease. Details of *F* and *F*_0_, along with permutation tests and 95% confidence intervals for pOR, pRR and pChi are in Appendix I.

### Aggregate high risk from significant GxG interactions into risk scores

Assume a study investigates a total of *N* subjects. For the *n*^*th*^ (*n* = 1, 2, ⋯, *N*) subject, an aggregated *k–*way epistasis enriched risk score, *R*(*k,n*), is defined by

(4) $$\{R\left(k,n\right)=\sum_{i=1}^{\left(\begin{array}{l} M\\ k \end{array}\right)}\left[I\left\{\text{Pvalu}e_{i}<\hat{\alpha }\right\}\sum_{j=1}^{3^{k}}\left(I\left\{n\in C_{\text{ij}}\right\}I\left\{X_{\text{ij}}/\left(X_{\text{ij}}+Y_{\text{ij}}\right)>p_{i0}\right\}\right)\right]\;\;$$

(5) $$\{\hat{\alpha }=\underset{0\leq \alpha \leq 0.05}{\text{arg}\;\text{max}}\left\{\text{AUC}|\alpha \right\}\;$$

In equation (4), we use the indicator variable, $I\left\{\text{Pvalu}e_{i}<\hat{\alpha }\right\}$, to remove the *i*^*th*^ non-significant GxG interaction from further calculation of risk scores. For the remaining significant GxG interactions, the indicator function, *I*{*n* ∈ *C*_*ij*_}*I*{*X*_*ij*_/(*X*_*ij*_ + *Y*_*ij*_) >*p*_*i*0_}, assigns 1 if the *n*^*th*^ subject carries a high predisposing risk genotypic combination and 0 if the *n*^*th*^ subject carries a low predisposing risk genotypic combination. More specifically, *I*{*X*_*ij*_/(*X*_*ij*_ + *Y*_*ij*_) >*p*_*i*0_} indicates whether the *j*^*th*^ genotypic combination has high predisposing risk and *I*{*n* ∈ *C*_*ij*_} checks whether the *n*^*th*^ subject carries the *j*^*th*^ genotypic combination. Each subject’s risk scores are then summed over all 3^*k*^genotypic combinations and all *k–*way GxG interactions to obtain an aggregated *k–*way epistasis enriched risk score, *R*(*k,n*), for the *n*^*th*^ subject.

Finally, the epistasis enriched risk score, *R*(*k,n*), is treated as a diagnostic test for the disease. One can consider adding up the predisposing scores with p < 0.05 from these interactions. Our experience has been that, in many cases, *α*=0.05 is an arbitrary cutoff for p-values and some GxG interactions with p-value < 0.05 might have low predictive ability. Therefore, a receiver operating characteristic (ROC) curve is constructed, and the area under the ROC curve (AUC) provides an overall evaluation of the epistasis enriched risk score’s ability to predict disease susceptibility. Instead of using an arbitrary cutoff *α*=0.05, we propose to select $\hat{\alpha }$ that maximizes the AUC of ROC curves. Choosing $\hat{\alpha }=\underset{0\leq \alpha \leq 0.05}{\text{arg}\;\text{max}}\left\{\text{AUC}|\alpha \right\}\;$ for $\hat{\alpha }\leq 0.05$ may help focusing on a modest number of summands that are most conducive to correct classification (arising from the strongest GxG interactions), rather than diluting the epistasis enriched risk score *R*(*k,n*) by a large number of summands that are not as conducive to correct classification (arising from comparatively weaker GxG interactions).

### Construct multiple GxG interactions into an epistasis enriched network

**Figure 2:**
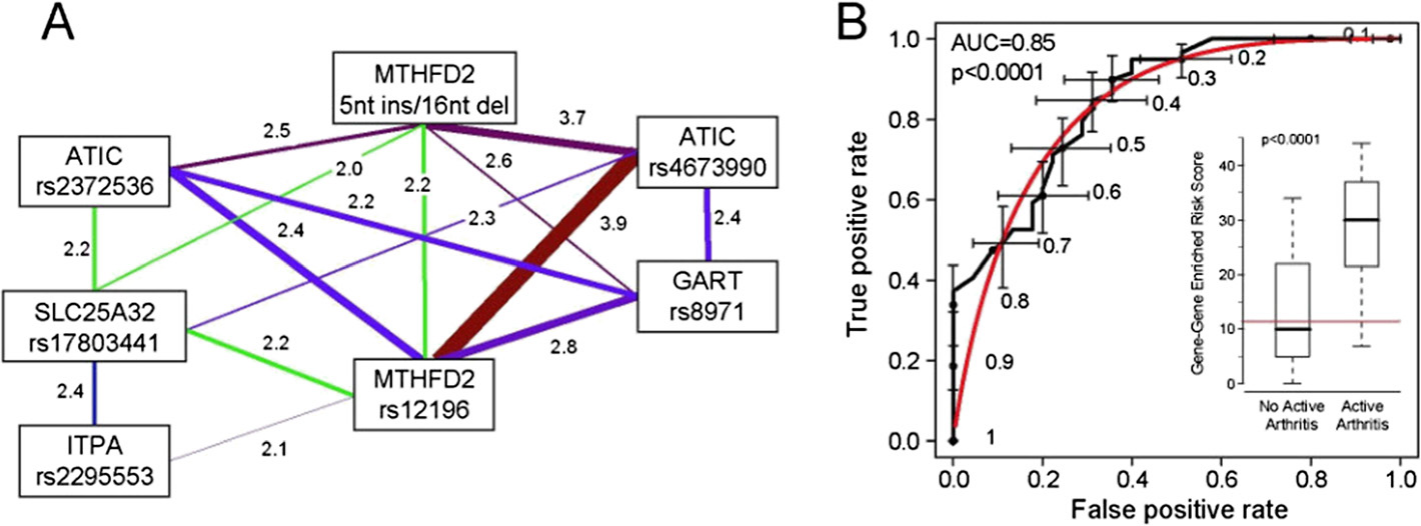
**Panel A: gene-gene interaction network of 7 SNPs associated with susceptibility to active arthritis.** Significant GxG interactions (FDR<0.05) are connected by line, and the strength of interaction is labeled with pOR. **A** larger pOR indicates a stronger interaction (thicker line) while a smaller pOR value indicates a relatively milder interaction (thinner lines). Panel **B**: ROC curve for risk scores derived from 82 significant two-locus interactions pooled from 34 SNPs. The risk score is significantly higher in patients with a poor response to MTX (active arthritis; inset).

The significant GxG interactions used to define the epistasis enriched risk score are collectively referred to as an epistasis enriched network (Figure 2A). Genes involved in one or more significant interactions appear as nodes in a radial graph. Pairs of genes sharing significant interactions are connected by lines. Each line is labeled with the corresponding pOR, and the line thickness may be chosen to accentuate the strongest pORs.

In summary, the A-MDR method has not only replaced the dichotomous predisposing risk factor with a continuous predictive variable, *R*(*k,n*), but has done so by integrating numerous significant GxG interactions into an epistasis enriched network that may more adequately explain the susceptibility to complex diseases. Epistasis enriched risk scores may also be accumulated over GxG interactions from multiple dimensions. For instance, we can accumulate both two-way and three-way GxG interactions. Or further consider accumulating one way main effects and up to *k–*way GxG interactions. The feasibility of these extensions needs to be assessed by future studies.

## Empirical assessment

Extensive Monte Carlo simulations were performed to assess the performance of pOR, pRR and pChi and compare them to the original MDR in unrelated case–control studies. To avoid subjective selections of models in favor of our methods, we report the power and type I error simulation similar to the models previously assessed by [26]. In each model, we simulated five SNPs with common homozygote (AA), heterozygote (Aa) and rare homozygote (aa). The minor allele frequencies (MAF) in each model were set to be 0.5 and 0.25 respectively and the genotypes were generated according to proportional expectations under Hardy-Weinberg equilibrium and linkage equilibrium. Equal numbers of affected and unaffected subjects were generated based on penetrance functions given in Table 2. Let *D*_=1_ indicate the onset of disease and *l*1,*l*2...,*l*5 stand for five loci, *P*_*l*1×*l*2_ and *P*_*l*4×*l*5_ be penetrance functions from loci 1x2 and loci 4x5 respectively as listed in Table 2. Our simulations comprised three major scenarios:

A) Existence of only one two-way interaction in loci 1x2 associated with disease susceptibility while the remaining loci were unrelated to the outcome variable, i.e. *P*(*D* = 1|*l*1, *l*2) = *p*_*l*1 × *l*2_.

B) Genetic heterogeneity models where a proportion of affected subjects are linked with interactions between loci 1 and 2 and the rest of affected subjects are linked with interactions between loci 4 and 5, i.e.

$$\begin{array}{ll} \;P\left(D\right) & \left(=1,C|l1,l2,l4,l5\right)=P\left(D=1|l1,l2,l4,l5,C=0\right)\times P\left(C=0\right)\\ & +P\left(D=1|l1,l2,l4,l5,C=1\right)\times P\left(C=1\right)=P\left(D=1|l1,l2\right)\times P\left(C=0\right)\\ & +P\left(D=1|l4,l5\right)\times P\left(C=1\right)=\gamma_{1}p_{l1\times l2}+\gamma_{2}p_{l4\times l5} \end{array}$$

where *γ*_1_≥0,*γ*_2_≥0 and *γ*_1_+*γ*_2_=1. The latent binary variable *C* labels the source of genetic variation where *C*=0 indicates that disease is related to loci 1x2 with P(C=0)=*γ*_1_ and *C*=1 indicates that disease is related to loci 4x5 with *P*(*C*=1)=γ_2_. In this study, we consider balanced genetic heterogeneity models with γ_1_=γ_2_=0.5 and unbalanced genetic heterogeneity models with *γ*_1_=0.7 and *γ*_2_=0.3.

C) Additive models with two pairs of loci jointly contributing to disease susceptibility. Let *D*_*l*1×*l*2_=1 denote the onset of disease due to the penetrance *P*_*l*1×*l*2_ from loci 1*2 and *D*_*l*4×*l*5_=1 due to the penetrance *P*_*l*4×*l*5_ from loci 4*5. The susceptibility function is given by

$$\begin{array}{ll} \;P(D=1|l1,l2,l4,l5) & =P\left(\left(D_{l1\mathrm{xl}2}=1\right)\cup \left(D_{l4\mathrm{xl}5}=1\right)|l1,l2,l4,l5\right)=P\left(D_{l1\mathrm{xl}2}=1|l1,l2,l4,l5\right)\\ & +P\left(D_{l4\mathrm{xl}5}=1|l1,l2,l4,l5\right)-P\left(\left(D_{l1\mathrm{xl}2}=1\right)\cap \left(D_{l4\mathrm{xl}5}=1\right)|l1,l2,l4,l5\right)\\ & =P\left(D_{l1\mathrm{xl}2}=1|l1,l2\right)+P\left(D_{l4\mathrm{xl}5}=1|l4,l5\right)-P\left(D_{l1\mathrm{xl}2}=1|l1,l2\right)\\ & \times P\left(D_{l4\mathrm{xl}5}=1|l4,l5\right)=p_{l1\times l2}+p_{l4\times l5}-p_{l1\times l2}p_{l4\times l5} \end{array}$$

**Table 2:** Simulated gene-gene interaction models with varying penetrance functions and minor allele frequencies

		**Model 1**			**Model 2**		
	BB	Bb	bb		BB	Bb	bb
AA	0	0.1	0	AA	0	0	0.1
Aa	0.1	0	0.1	Aa	0	0.05	0
aa	0	0.1	0	aa	0.1	0	0
		(A) MAF=0.5				(B) MAF=0.5	
		Model 3				Model 4	
	BB	Bb	bb		BB	Bb	bb
AA	0.08	0.07	0.05	AA	0.09	0.05	0.02
Aa	0.1	0	0.1	Aa	0.08	0.09	0.01
aa	0.03	0.1	0.04	aa	0.03	0.01	0.03
		(C) MAF=0.25				(D) MAF=0.25	

To assess the power of the A-MDR method, we randomly generated 100 sets of data in the above described scenarios and performed the MDR and A-MDR tests for each random sample. The power is the percentage of rejection of null hypothesis for the loci with a GxG interaction. Type I error is the percentage of rejection of null hypothesis when the simulated loci have no GxG interaction.

As shown in Table 3, the Type I errors of all tests were under the nominal rate of 5%. When only loci 1 and 2 had an interaction (Table 3A), all five measures had strong power to detect GxG interactions in most models except that the power of MDR dropped to 0.46 in model 3 with n=300, which could be enhanced by increasing sample size. We next simulated genetic heterogeneity (Table 3B) with 0.5/0.5, 0.7/0.3 proportions of subjects affected by a mixture model of epistasis from loci 1x2 and loci 4*5. We noticed that MDR lost power to detect interactions with weaker effects. The power was not recovered with increased sample sizes. We last examined the additive models (Table 3C) where susceptibility increases jointly through loci 1x2 and loci 4x5. MDR has low power to detect both pairs of GxG interactions. All this evidence suggests that the proposed A-MDR might be a better choice for detecting complex GxG interactions, especially when multiple GxG interactions are cumulatively contributing to a phenotype.

**Table 3:** Power and type I error assessment

A) Only Locus1*2 has an interaction	**Model 1 (n=300)**		**Model 2 (n=300)**		**Model 3 (n=300)**		**Model 4 (n=300)**	
	**Power**	**Type I**	**Power**	**Type I**	**Power**	**Type I**	**Power**	**Type I**
A-MDR pOR	1	0.01	1	0.01	0.75	0.01	1	0.03
A-MDR pRR	1	0	1	0.01	0.71	0.01	1	0.03
A-MDR pChi	1	0.01	1	0	0.86	0.03	1	0
MDR	1	0	1	0	0.46	0.02	1	0
A) Only Locus1*2 has an interaction	Model 1 (n=400)		Model 2 (n=400)		Model 3 (n=400)		Model 4 (n=400)	
	Power	Type I	Power	Type I	Power	Type I	Power	Type I
A-MDR pOR	1	0	1	0	0.86	0	1	0.02
A-MDR pRR	1	0	1	0	0.87	0	1	0.03
A-MDR pChi	1	0.02	1	0.01	0.95	0.01	1	0.02
MDR	1	0	1	0	0.68	0.01	1	0
**B) Genetic heterogeneity *γ***_**1**_**=*γ***_**2**_**=0.5**	Model 1	Model 2	Model 1	Model 2	Model 3	Model 4	Model 3	Model 4
	n=400 Power		n=800 Power		n=400 Power		n=800 Power	
A-MDR pOR	0.98	1	1	1	0.37	0.91	0.7	1
A-MDR pRR	0.99	1	1	1	0.35	0.78	0.68	1
A-MDR pChi	0.99	1	1	1	0.31	0.99	0.72	1
MDR	0.12	0.75	0.08	0.78	0	0.69	0	0.92
**B) Genetic heterogeneity γ**_**1**_**=0.7,γ**_**2**_**=0.3**	Model 1	Model 2	Model 1	Model 2	Model 3	Model 4	Model 3	Model 4
	n=400 Power		n=800 Power		n=400 Power		n=800 Power	
A-MDR pOR	1	0.35	1	0.98	0.55	0.35	0.92	0.78
A-MDR pRR	1	0.25	1	0.97	0.55	0.3	0.91	0.74
A-MDR pChi	1	0.5	1	0.99	0.6	0.49	0.96	0.86
MDR	1	0	0.99	0	0.14	0.21	0.14	0.38
**C) Additive Models**	Model 1	Model 2	Model 1	Model 2	Model 3	Model 4	Model 3	Model 4
	n=400 Power		n=800 Power		n=400 Power		n=800 Power	
A-MDR pOR	1	0.72	1	1	0.6	0.16	0.93	0.61
A-MDR pRR	1	0.69	1	0.98	0.61	0.14	0.91	0.55
A-MDR pChi	1	0.79	1	1	0.71	0.29	0.98	0.76
MDR	0.91	0.02	0.99	0.01	0.26	0.14	0.43	0.12

(Within each cell, loci 1x2 is listed in the first column and loci 4x5 is in the second column).

## Application to genotyping data

Juvenile Idiopathic Arthritis (JIA) is one of the most common chronic diseases of childhood, affecting an estimated 300,000 children in the U.S. alone, and is an important cause of morbidity and disability in children. Although methotrexate (MTX) is the most commonly used second-line anti-inflammatory agent used to treat JIA worldwide, this antifolate prodrug has shown considerable inter-individual variability in clinical response and adverse reactions. Thus far, variables investigated as potential useful predictors of response and toxicity in patients taking MTX, which is used alone and as an “anchor drug” for many rheumatic conditions, have not been clearly associated with outcomes. The effect of individual genetic SNP variation within the folate pathway upon MTX response has been investigated in several studies in adult rheumatoid arthritis (RA) and a few studies in JIA with conflicting results. To elucidate the genetic architecture impacting the efficacy of MTX, 34 SNPs from 19 folate pathway genes were measured in 104 subjects. Response-defined as the absence of active arthritis (swelling not due to bony enlargement or, if no swelling was present, limitation of motion accompanied by either pain on motion or tenderness, not due to trauma or explained by prior joint damage [27]-was determined for these subjects. Information pertaining to the 34 SNPs is listed in Table 4; demographic and clinical characteristics of the subjects were described by Becker and colleagues [15]. After being on a stable dose and route of MTX for at least 3 months, 56.7% of the patients (59 out of 104) still had at least 1 active (i.e., swollen or tender) joint. The presence of active arthritis was the outcome variable, and represented an incomplete response to MTX. By definition, the absence of active arthritis-no joint involvement-was considered a positive response to MTX treatment.

**Table 4:** List of 34 SNPs from 18 candidate genes in the folate pathway

**Gene**	**RS#**	**Gene**	**RS#**
**ABCG2**	rs7699188	**GGH**	rs3758149
	−15846A>C		rs11545078
	rs35252139	**ITPA**	rs2295553
	rs35229708	**MTHFD1**	rs2236225
	rs55930652	**MTHFD2**	5nt ins/16nt del
**ADORA2a**	rs2298383		rs56168672
	rs3761422		rs12196
	rs2267076	**MTHFR**	rs1801133
	rs2236624		rs1801131
**ATIC**	rs2372536		rs2274976
	rs12995526	**MTR**	rs1805087
	rs4673990	**MTRR**	rs1801394
**BHMT**	rs3733890	**SHMT-1**	rs1979277
**DHFR**	19 bp deletion	**SLC25A32**	rs17803441
	rs7387	**SLCO1B1**	Rs4149056
**GART**	rs8971	**SLC19A1**	rs1051266
**TSER**	rs34743033	**TYMS**	rs11280056

Standard logistic regression analysis did not identify significant main effects from SNPs or GxG interactions. This could primarily due to the complex and non-linear interactions among SNPs. For the rest article, the A-MDR and original MDR methods were applied to the MTX data to search for genetic predictors of response to MTX. Redundant SNPs and SNPs with no prediction of the phenotype were removed by the ReliefF algorithm [7]. A complete set of 34 SNPs and 7 filtered SNPs were analyzed respectively.

The original MDR analysis method was applied to obtain the one-locus, two-locus, and three-locus models with the highest validation accuracy in the original MDR. Two-locus interactions between genes *ATIC* and *MTHFD2* were significant in testing accuracy but not in CVC. The prediction accuracy from the optimal MDR model was 75%.

We then utilized pOR, pRR, and pChi to identify and characterize GxG interactions in the A-MDR analyses. Numerous two-locus GxG interactions were significantly associated with efficacy of MTX based on pOR, pRR, and pChi. We found that pChi flagged the most GxG interactions as significantly associated with efficacy of MTX. After we used the ReliefF algorithm to narrow down the list of candidate SNPs to 7, 15 pairs GxG interactions were flagged as significant after FDR correction. Table 5 lists pOR, pRR, and pChi along with 95% confidence intervals, unadjusted p-values, and FDR-adjusted p-values for two-locus GxG interactions among 7 filtered SNPs. For the most part, the three measures of GxG interactions (pOR, pRR, and pChi) yielded unadjusted p-values that were in qualitative agreement. The epistasis-enriched network based on the 15 significant two-locus GxG interactions from seven SNPs in five genes appears in Figure 2A.

**Table 5:** Two-locus GxG interactions among 7 SNPs assessed by A-MDR

**Two-locus GxG Interactions**	**pOR**	**p-value**	**FDR**	**pRR**	**p-value**	**FDR**	**pChi**	**p-value**	**FDR**
ATIC rs4673990+ MTHFD2 rs12196	3.9 (1.6–13.1)	0.001*	0.013*	1.9 (1.2-4.1)	0.001*	0.027*	6.1 (1.8-21.1)	<.001*	0.002*
ATIC rs4673990 + MTHFD2 5nt ins/16nt del	3.7 (1.5-12.8)	0.001*	0.013*	1.8 (1.1-3.6)	0.003*	0.032*	6.0 (1.6-22.0)	<.001*	0.002*
MTHFD2 rs12196 + GART rs8971	2.8 (1.1-8.8)	0.013*	0.069	1.6 (0.9-3.4)	0.016*	0.050	5.5 (1.3-20.7)	0.001*	0.009*
MTHFD2 5nt ins/16nt del + GART rs8971	2.6 (1.0-7.9)	0.017*	0.069	1.5 (0.9-2.7)	0.017*	0.050	5.0 (1.1-20.6)	0.002*	0.011*
ATIC rs4673990 + GART rs8971	2.5 (1.1-8.8)	0.027*	0.071	1.4 (0.9-2.4)	0.038*	0.088	4.7 (1.1-19.2)	0.009*	0.019*
ATIC rs2372536 + MTHFD2 5nt ins/16nt del	2.5 (1.0-8.3)	0.017*	0.069	1.6 (1.0-3.6)	0.006*	0.041*	5.0 (1.1-17.6)	0.011*	0.020*
ATIC rs2372536 + MTHFD2 rs12196	2.4 (0.9-8.0)	0.021*	0.069	1.4 (1.0-3.0)	0.033*	0.088	5.6 (1.2-18.3)	0.008*	0.019*
ATIC rs2372536 + GART rs8971	2.2 (0.8-6.5)	0.032*	0.074	1.5 (0.9-2.8)	0.015*	0.050	4.5 (0.8-28.2)	0.012*	0.020*
SLC25A32 rs17803441 +ITPA rs2295553	2.4 (0.8-7.5)	0.023*	0.069	1.6 (1.0-2.8)	0.013*	0.050	4.2 (0.8-17.9)	0.005*	0.019*
ATIC rs4673990 + SLC25A32 rs17803441	2.3 (0.8-8.2)	0.039*	0.081	1.4 (0.8-2.2)	0.061	0.106	4.9 (0.9-30.9)	0.009*	0.019*
MTHFD2 rs12196 + ITPA rs2295553	2.1 (0.9-7.1)	0.047*	0.090	1.4 (0.8-2.1)	0.065	0.106	4.2 (1.0-16.0)	0.025*	0.034*
ATIC rs4673990 + ITPA rs2295553	2.3 (0.7-7.1)	0.074	0.101	1.4 (0.7-2.2)	0.076	0.106	3.7 (0.9-13.2)	0.045*	0.059
MTHFD2 rs12196 + SLC25A32 rs17803441	2.2 (0.7-6.9)	0.060	0.097	1.4 (0.8-2.3)	0.067	0.106	5.2 (0.9-27.2)	0.012*	0.020*
ATIC rs2372536 + SLC25A32 rs17803441	2.2 (0.7-6.1)	0.056	0.097	1.3 (0.9-2.7)	0.062	0.106	6.6 (0.9-37.9)	0.008*	0.019*
MTHFD2 5nt ins/16nt del + MTHFD2 rs12196	2.0 (0.9-7.0)	0.074	0.101	1.3 (0.9-2.2)	0.088	0.115	5.2 (0.8-33.1)	0.008*	0.019*
MTHFD2 5nt ins/16nt del +LC25A32 rs17803441	2.0 (0.6-5.9)	0.077	0.101	1.3 (0.9-3.3)	0.073	0.106	4.2 (0.7-25.5)	0.015*	0.023*
MTHFD2 5nt ins/16nt del + ITPA rs2295553	1.9 (0.8-6.4)	0.114	0.139	1.3 (0.9-2.6)	0.129	0.160	3.9 (0.8-14.6)	0.076	0.094
ATIC rs2372536 + ITPA rs2295553	1.9 (0.8-6.9)	0.119	0.139	1.3 (0.9-2.2)	0.182	0.201	3.9 (0.8-13.3)	0.091	0.101
GART rs8971 + ITPA rs2295553	1.9 (0.8-8.9)	0.186	0.195	1.3 (0.9-3.6)	0.217	0.228	4.7 (0.8-16.1)	0.124	0.130
ATIC rs2372536 + ATIC rs4673990	1.8 (0.7-5.3)	0.131	0.145	1.2 (0.8-2.0)	0.161	0.188	4.0 (0.7-23.3)	0.089	0.101
GART rs8971 + SLC25A32 rs17803441	1.7 (0.4-9.9)	0.227	0.227	1.2 (0.7-5.7)	0.296	0.296	3.5 (0.4-19.1)	0.156	0.156

(Three measures of GxG interactions [pOR, pRR, and pChi] along with 95% confidence intervals [CI] were obtained for each two-locus GxG interaction. The p-value columns list unadjusted p-values, while the FDR columns list p-values after adjustment for multiple comparisons. Significant GxG interactions, as judged by p-value or FDR less than 0.05, are labeled with asterisks. The first GxG interaction in the table was the optimal two-locus GxG interactions identified by the original MDR method with significant testing accuracy but insignificant CVC).

Another goal of our A-MDR analysis was to integrate numerous significant GxG interactions into a continuous epistasis enriched risk score for the prediction of which patients would have active arthritis despite MTX treatment. A higher epistasis enriched risk score would indicate that a patient carried more high-risk genotypic combinations in loci with significant GxG interactions, and vice versa. To compare prediction accuracies based on the number of candidate SNPs as well as the presence or absence of adjustment for multiple comparisons, we generated epistasis enriched risk scores from 82 significant GxG interactions from 34 SNPs (Figure 2B).

Subjects with persistent active arthritis had significantly higher mean and median epistasis enriched risk scores compared to subjects without active arthritis (p < 0.0001). When 82 GxG interactions from 34 SNPs with unadjusted p-values < 0.0167 were used to generate epistasis enriched risk scores (Figure 2B boxplot inset), these scores ranged from 0 to 44. A higher risk score suggests that a subject is less likely to respond favorably to MTX treatment. The ROC curve assessing the overall ability of the epistasis enriched risk score to distinguish between subjects with active joints and subjects without active joints had 85% area under the curve (p < 0.0001). (The 0.0167 cutoff for unadjusted p-values was chosen to maximize this area.) We correctly classify 82% of the subjects if we predict that those with epistasis enriched risk scores above 11.5 have active joints and that those with epistasis enriched risk scores below 11.5 do not have persistent joint involvement.

Examination of the five genes in the 15-interaction model presented in Figure 2A reveals a testable hypothesis for future studies. All genes fall within a pathway leading to purine biosynthesis and adenosine formation: *SLC25A32* transports folates from the cytoplasm to mitochondria; *MTHFD2* is a component of the mitochondrial folate pathway that produces one-carbon donors in the form of formate (10-formyl-tetrahydrofolate) exclusively to support *de novo* purine biosynthesis; and *ITPA, ATIC*, and *GART* are involved in purine biosynthesis. Thus, all genes map to a core pathway associated with adenosine accumulation, which is considered to be a mechanism of action of MTX that contributes to response in JIA and Rheumatoid Arthritis.

## Discussion

In this work, we have proposed an Aggregated Multifactor Dimensionality Reduction (A-MDR) model to elucidate complex and non-linear genetic associations contributing to disease risk and variability in response to treatment. The proposed method is innovative in three important ways: 1) a continuous GxG enriched risk score is generated to replace the dichotomous risk factor in prediction of susceptibility to disorders; 2) new measures of gene-gene interaction using pOR, pRR, and pChi along with p-values and confidence intervals are proposed to detect and characterize *multiple* gene-gene interactions; and, 3) a radial network is generated to depict patterns of epistasis. This approach allows for prediction on not just a single interactive model, which is important given the growing appreciation in human genetics for the accumulative impact of a large number of variants with low effect size [28]. By pooling moderate and inter-related genetic contributors together, the A-MDR model becomes robust and predictive of complex traits. In addition to GxG interactions, the A-MDR can also be applied to model gene-environment interactions where environmental risk factors such as smoking, alcohol consumption, exercise, and diet can be incorporated into multi-factorial models.

The original MDR model selects an optimal multi-factorial (SNP) combination for each two-way, three-way or higher order interaction. When multiple genes function together in a pathway, the original MDR is prone to overlook genes with weaker signals and lose power for selecting one optimal GxG interaction in cross-validation. For the MTX data, the optimal two-locus interaction detected by the original MDR among 7 candidate SNPs was *ATIC* (rs4673990) + *MTHFD2* (rs12196) with testing accuracy of 0.73 (p=0.0005). However, there exist other pairs of interactions with comparable accuracy. As a result, CVC, which measures the percentage of times that an optimal GxG interaction is selected when splitting the training and validation sets randomly, was not significant (CVC=8/10, p=0.2700). Our A-MDR analysis in Table 5 identified 15 pairs of two-locus interactions. When multiple GxG interactions with *bio-equivalent* effects are involved in epistasis, the original MDR will select an optimal model, by chance and lose some of the real pathway-based signals. The recent extended MDR methods, including OR-MDR [17], LM-MDR [18] and G-MDR [19], adopt the same strategy of selecting one optimal GxG interaction as does the original MDR, which means they have the same limitations.

A continuous GxG enriched risk score is another major distinction between A-MDR and all the majority of existing MDR models, in which a binary risk factor is utilized to predict the outcome variable. For *M*-way interactions, the existing MDR models classify *~3*^*M*^ genotypic combinations as either high-risk or low-risk. A-MDR evolves from the traditional MDR outputs to the predisposing risk scores and epistasis based network as shown in Figure 2.

Another important result of the simulation experiments is the potential of A-MDR to detect models that include genetic heterogeneity. Previous work with the original MDR has shown that heterogeneity is disastrous when using MDR to detect interactions [26][29]. Because of the use of the continuous enrichment score, A-MDR is less impacted by heterogeneity in the enclosed simulations. Further evaluation of this initial result with expanded simulations and real data applications will be an important next step.

We explore a radial network (Figure 2A) to depict patterns of epistasis. From the systems biology perspective, genetic variants might jointly impact the disease susceptibility and response to treatment. The gene-gene interaction network reveals intriguing information when interpreted in the context of what we know about the folate pathway and the effect that MTX has upon the disruption of this pathway as it relates to arthritis. ATIC and MTHFD2 were the two genes with the strongest interaction, and it is of interest to note that the genes included in the model (Figure 2A) include a transporter involved in folate uptake into mitochondria, SLC25A32, and the bifunctional methylenetetrahydrofolate dehydrogenase-cyclohydrolase MTHFD2, a key constituent of the mitochondrial folate pathway. The mitochondrial folate pathway is responsible for the generation of formate (in the form of 10-formylTHF) specifically to support purine biosynthesis, represented by ATIC, GART, and ITPA. The anti-inflammatory effect of low-dose MTX used to treat JIA and RA is thought to be due the anti-inflammatory effects of adenosine, formed as a consequence of the inhibitory effects of MTX on amino-imidazole carboxamide ribonucleotide (AICAR) transformylase (gene name, *ATIC*), which promotes the accumulation of AICAR ribotide, inhibiting adenosine deaminase and leading to a build up of adenosine, a potent anti-inflammatory agent [30]. A disruption of this process may result in a decreased anti-inflammatory effect of the drug. Therefore, the combined effect of SNPs in *ATIC* and *MTHFD2* may indeed yield a more clinically apparent result by altering the anti-inflammatory effects of methotrexate. There is a potential to apply the proposed method to GWAS study by dissecting SNPs into pathways in order to detect GxG interactions in GWAS pathways. The major computational challenges from the proposed A-MDR and other approach in MDR framework are in the generation of p-values for MDR. MDR permutation computing time is largely dependent on the dimension of data sets. In other words, the computing time increases as the number of SNPs and/or the number of subjects increases. Several works have been devoted to improve the efficiency and shorten the computing time in MDR analysis in high-throughput data [5,31,32]. We will defer interested readers to the corresponding citations for computing issues in high-throughput MDR analysis. These computational limitations make our strategy appropriate in large scale candidate gene studies, but may be limited in application to genome-wide association studies until further improvements in computing speed are realized or very large-scale computing resources are available.

In summary, bioinformatics challenges remain in detecting and modeling epistasis in complex biological traits. We have developed a new A-MDR framework to interpret complex genetic variation and have proposed predicting an outcome using a continuous risk factor. Several other extensions and modifications of the original MDR have been proposed in the literature. Incorporation of valuable features from other MDR extension models into the A-MDR framework is worth further investigation. Prospective studies and validation in independent samples are needed to assess reliability of the A-MDR model’s predictive ability. Tools for statistical inference, including asymptotic distributions of the proposed test statistics, need to be developed to save computing time and improve reliability.

## Appendix

Appendix I. Justification, 95% confidence intervals and permutation tests of pOR, pRR and pChi.

Since the predisposing risk factor (Table 1) is conditioned on the naïve Bayes classifier, standard inference procedures based on normal or chi-square asymptotic distributions with 1 degree of freedom do not apply to the numerators in (1)-(3), which are the unadjusted odds ratio (OR), relative risk (RR) and Chi-square statistics (Chi). As a result, 95% confidence intervals of OR and RR are often greater than 1 under H_0_. To address this issue, we propose pOR, pRR and pChi by taking the null distribution of unadjusted statistics into account. Let *x*=*pOR*,*pRR*, or *pChi*, *F*(*x*) be the cumulative distribution function of the corresponding statistic under the alterative hypothesis (GxG interaction present) *F*_0_(*x*),be the cumulative distribution function of the corresponding statistic under the null hypothesis (GxG interaction absent) and *F*_0_^− 1^(*x*) be the inverse function of *F*_0_(*x*). The corrected pOR, pRR and pChi are then defined by $\frac{x}{F_{0}^{-1}\left(F\left(x\right)\right)}$. Under *H*_0_, *F*(*x*)=*F*_0_(*x*), so pOR, pRR and pChi should equal $\frac{x}{F_{0}^{-1}\left(F\left(x\right)\right)}=\frac{x}{F_{0}^{-1}\left(F_{0}\left(x\right)\right)}=1$. This adjustment will ensure the insignificant GxG interactions to have 95% confidence interactions cross 1 under Under *H*_0_. In this work, we evaluated pOR, pRR and pChi using a full data set while these methods can also be evaluated under the cross validation scheme typically used in MDR.

The functions *F*(*x*) and *F*_0_(*x*) can be estimated by the corresponding empirical distribution function. Permutation is applied to estimate *F*_0_(*x*) by reshuffling the relationship between SNPs and a phenotype, where SNPs for each individual in a system are maintained as a vector to preserve their correlation structure. For each permutation, we generated odds ratio (OR), relative risk (RR) and chi-square test statistic (Chi). Jackknife re-sampling was applied to estimate *F*(*x*) by generating random subsets of data, where 80% to 90% of subjects were randomly selected. SNPs and the phenotype from each subject are maintained as a vector to preserve the association between SNPs and the phenotype. Denote the OR, RR and Chi statistic from permutation or re-sampling as *x*_1_,*x*_2_,…*x*_*B*_ where *B* is the number of permutation or resampling. The null distribution function *F*_0_(*x*) and *F*(*x*) can be estimated by $B^{-1}\sum_{i=1}^{B}I_{\left\{x_{i}\leq x\right\}}$. The 95% confidence interval pOR, pRR and pChi can be obtained by resampling. Denote the pOR, pRR and pChi statistics from resampling or permutation as *z*_1_,*z*_2_…,*z*_B_ then the 95% confidence interval pOR, pRR and pChi is the interval from 2.5 to 97.5 percentile of *z*_1_,*z*_2_…,*z*_B_ from resampling. The p-value for pOR, pRR and pChi for the *i*^*th*^ GxG interaction, denoted by *Pvalue*_*i*_, will be calculated by the permutation testing, i.e. $\text{Pvalu}e_{i}=B^{-1}\sum_{i=1}^{B}I_{\left\{z<z_{i}\right\}}$ where *z*_1_,*z*_2_…,*z*_B_ are calculated from permutation samples and *z* is the pOR, pRR and pChi statistic calculated from the current data.

## Competing interests

There are no competing interests to this work.

## Authors’ contributions

HD conceived of the study. RC and AMR aided in study design and statistical method. HD performed the simulations and data analysis. MB and SL performed the clinical data collection, genotyping and interpretation of case study findings. All authors contributed to the manuscript writing. All authors read and approved the final manuscript.

## References

[B1] Moore JH Detecting, characterizing, and interpreting nonlinear gene-gene interactions using multifactor dimensionality reduction Adv Genet 2010 72 101 116 21029850 10.1016/B978-0-12-380862-2.00005-9

[B2] Cantor RM Lange K Sinsheimer JS Prioritizing GWAS results: a review of statistical methods and recommendations for their application Am J Hum Genet 2009 86 1 6 22 20074509 10.1016/j.ajhg.2009.11.017 PMC2801749

[B3] Ritchie MD Hahn LW Roodi N Bailey LR Dupont WD Parl FF Moore JH Multifactor-dimensionality reduction reveals high-order interactions among estrogen-metabolism genes in sporadic breast cancer Am J Hum Genet 2001 69 1 138 147 10.1086/321276 11404819 PMC1226028

[B4] Hahn LW Ritchie MD Moore JH Multifactor dimensionality reduction software for detecting gene-gene and gene-environment interactions Bioinformatics 2003 19 3 376 382 10.1093/bioinformatics/btf869 12584123

[B5] Bush WS Dudek SM Ritchie MD Parallel multifactor dimensionality reduction: a tool for the large-scale analysis of gene-gene interactions Bioinformatics 2006 22 17 2173 2174 10.1093/bioinformatics/btl347 16809395 PMC4939609

[B6] Winham SJ Motsinger-Reif AA An R package implementation of multifactor dimensionality reduction BioData Min 2011 4 1 24 10.1186/1756-0381-4-24 21846375 PMC3177775

[B7] Robnik-Siknja M Kononeko I Theoretical and empirical analysis of RelifF and RReliefF Mach Learn 2003 53 23 69 10.1023/A:1025667309714

[B8] Dai H Bhandary M Becker ML Leeder SJ Gaedigk R Motsinger-Reif AA Global tests of p-values for multifactor dimensionality reduction models in selection of optimal number of target genes BioData Min 2012 5 1 3 10.1186/1756-0381-5-3 22616673 PMC3508622

[B9] Motsinger AA Ritchie MD The effect of reduction in cross-validation intervals on the performance of multifactor dimensionality reduction Genet Epidemiol 2006 30 6 546 555 10.1002/gepi.20166 16800004

[B10] Hastie T Tibshirani R Friedman J The Elements of Statistical Learning: Data Mining, Inference, and Predication 2001 New York, USA: Springer

[B11] Good P Permutation Tests: A Practical Guide to Resampling Methods for Testing Hypotheses 2000 New York, USA: Springer

[B12] Moore JH Williams SM New strategies for identifying gene-gene interactions in hypertension Ann Med 2002 34 2 88 95 10.1080/07853890252953473 12108579

[B13] Andrew AS Karagas MR Nelson HH Guarrera S Polidoro S Gamberini S Sacerdote C Moore JH Kelsey KT Demidenko E DNA repair polymorphisms modify bladder cancer risk: a multi-factor analytic strategy Hum Hered 2008 65 2 105 118 10.1159/000108942 17898541 PMC2857629

[B14] Cho YM Ritchie MD Moore JH Park JY Lee KU Shin HD Lee HK Park KS Multifactor-dimensionality reduction shows a two-locus interaction associated with Type 2 diabetes mellitus Diabetologia 2004 47 3 549 554 10.1007/s00125-003-1321-3 14730379

[B15] Becker ML Gaedigk R van Haandel L Thomas B Lasky A Hoeltzel M Dai H Stobaugh J Leeder JS The effect of genotype on methotrexate polyglutamate variability in juvenile idiopathic arthritis and association with drug response Arthritis Rheum 2011 63 1 276 285 10.1002/art.30080 20954192

[B16] Dervieux T Wessels JA van der Straaten T Penrod N Moore JH Guchelaar HJ Kremer JM Gene-gene interactions in folate and adenosine biosynthesis pathways affect methotrexate efficacy and tolerability in rheumatoid arthritis Pharmacogenet Genomics 2009 19 12 935 944 10.1097/FPC.0b013e32833315d1 19858780

[B17] Chung Y Lee SY Elston RC Park T Odds ratio based multifactor-dimensionality reduction method for detecting gene-gene interactions Bioinformatics (Oxford England) 2007 23 1 71 76 10.1093/bioinformatics/btl557 17092990

[B18] Lee SY Chung Y Elston RC Kim Y Park T Log-linear model-based multifactor dimensionality reduction method to detect gene gene interactions Bioinformatics (Oxford England) 2007 23 19 2589 2595 10.1093/bioinformatics/btm396 17872915

[B19] Lou XY Chen GB Yan L Ma JZ Zhu J Elston RC Li MD A generalized combinatorial approach for detecting gene-by-gene and gene-by-environment interactions with application to nicotine dependence Am J Hum Genet 2007 80 6 1125 1137 10.1086/518312 17503330 PMC1867100

[B20] Velez DR White BC Motsinger AA Bush WS Ritchie MD Williams SM Moore JH A balanced accuracy function for epistasis modeling in imbalanced datasets using multifactor dimensionality reduction Genet Epidemiol 2007 31 4 306 315 10.1002/gepi.20211 17323372

[B21] Calle ML Urrea V Vellalta G Malats N Steen KV Improving strategies for detecting genetic patterns of disease susceptibility in association studies Stat Med 2008 27 30 6532 6546 10.1002/sim.3431 18837071

[B22] Bush WS Edwards TL Dudek SM McKinney BA Ritchie MD Alternative contingency table measures improve the power and detection of multifactor dimensionality reduction BMC Bioinformatics 2008 9 238 10.1186/1471-2105-9-238 18485205 PMC2412877

[B23] Lou XY Chen GB Yan L Ma JZ Mangold JE Zhu J Elston RC Li MD A combinatorial approach to detecting gene-gene and gene-environment interactions in family studies Am J Hum Genet 2008 83 4 457 467 10.1016/j.ajhg.2008.09.001 18834969 PMC2561932

[B24] Mei H Cuccaro ML Martin ER Multifactor dimensionality reduction-phenomics: a novel method to capture genetic heterogeneity with use of phenotypic variables Am J Hum Genet 2007 81 6 1251 1261 10.1086/522307 17999363 PMC2276344

[B25] Moore JH Gilbert JC Tsai CT Chiang FT Holden T Barney N White BC A flexible computational framework for detecting, characterizing, and interpreting statistical patterns of epistasis in genetic studies of human disease susceptibility J Theor Biol 2006 241 2 252 261 10.1016/j.jtbi.2005.11.036 16457852

[B26] Ritchie MD Hahn LW Moore JH Power of multifactor dimensionality reduction for detecting gene-gene interactions in the presence of genotyping error, missing data, phenocopy, and genetic heterogeneity Genet Epidemiol 2003 24 2 150 157 10.1002/gepi.10218 12548676

[B27] Giannini EH Ruperto N Ravelli A Lovell DJ Felson DT Martini A Preliminary definition of improvement in juvenile arthritis Arthritis Rheum 1997 40 7 1202 1209 9214419 10.1002/1529-0131(199707)40:7<1202::AID-ART3>3.0.CO;2-R

[B28] Yang J Manolio TA Pasquale LR Boerwinkle E Caporaso N Cunningham JM de Andrade M Feenstra B Feingold E Hayes MG Genome partitioning of genetic variation for complex traits using common SNPs Nat Genet 2011 43 6 519 525 10.1038/ng.823 21552263 PMC4295936

[B29] Ritchie MD Edwards TL Fanelli TJ Motsinger AA Genetic heterogeneity is not as threatening as you might think Genet Epidemiol 2007 31 7 797 800 10.1002/gepi.20256 17654613

[B30] Cronstein BN Naime D Ostad E The antiinflammatory mechanism of methotrexate. Increased adenosine release at inflamed sites diminishes leukocyte accumulation in an in vivo model of inflammation J Clin Invest 1993 92 6 2675 2682 10.1172/JCI116884 8254024 PMC288465

[B31] Oki NO Motsinger-Reif AA Multifactor dimensionality reduction as a filter-based approach for genome wide association studies Front Genet 2011 2 80 22303374 10.3389/fgene.2011.00080 PMC3268633

[B32] Yang C Wan X He Z Yang Q Xue H Yu W The choice of null distributions for detecting gene-gene interactions in genome-wide association studies BMC Bioinformatics 2011 12 Suppl 1 S26 10.1186/1471-2105-12-S1-S26 21342556 PMC3044281

